# Case report: Lumpy skin disease in an endangered wild banteng (*Bos javanicus*) and initiation of a vaccination campaign in domestic livestock in Cambodia

**DOI:** 10.3389/fvets.2023.1228505

**Published:** 2023-08-03

**Authors:** Alice Porco, Sokha Chea, Sreyem Sours, Vonika Nou, Milou Groenenberg, Cain Agger, Sothyra Tum, Vanna Chhuon, San Sorn, Chamnan Hong, Ben Davis, Sharyn Davis, Sereyrotha Ken, Sarah H. Olson, Amanda E. Fine

**Affiliations:** ^1^Wildlife Conservation Society, Cambodia Program, Phnom Penh, Cambodia; ^2^General Directorate of Animal Health and Production, Phnom Penh, Cambodia; ^3^World Wide Fund for Nature, Phnom Penh, Cambodia; ^4^National Animal Health and Production Research Institute, Phnom Penh, Cambodia; ^5^Forestry Administration, Phnom Penh, Cambodia; ^6^Department of Freshwater Wetlands Conservation, Phnom Penh, Cambodia; ^7^Our Future Organization, Preah Vihear, Cambodia; ^8^Wildlife Conservation Society, Health Program, New York, NY, United States

**Keywords:** case report, lumpy skin disease, wildlife-livestock interface, vaccination, banteng, wildlife, conservation, intervention

## Abstract

We describe a case of lumpy skin disease in an endangered banteng in Cambodia and the subsequent initiation of a vaccination campaign in domestic cattle to protect wild bovids from disease transmission at the wildlife-livestock interface. Lumpy skin disease virus (LSDV) was first detected in domestic cattle in Cambodia in June of 2021 and rapidly spread throughout the country. In September 2021, a banteng was seen in Phnom Tnout Phnom Pok wildlife sanctuary with signs of lumpy skin disease. Scab samples were collected and tested positive for LSDV. Monitoring using line transect surveys and camera traps in protected areas with critical banteng and gaur populations was initiated from December 2021-October 2022. A collaborative multisector vaccination campaign to vaccinate domestic livestock in and around priority protected areas with banteng and gaur was launched July 2022 and a total of 20,089 domestic cattle and water buffalo were vaccinated with Lumpyvax^TM^. No signs of LSDV in banteng or gaur in Cambodia have been observed since this initial case. This report documents the first case of lumpy skin disease in wildlife in Cambodia and proposes a potential intervention to mitigate the challenge of pathogen transmission at the domestic-wildlife interface. While vaccination can support local livestock-based economies and promote biodiversity conservation, it is only a component of an integrated solution and One Health approach to protect endangered species from threats at the wildlife-livestock interface.

## Introduction

Lumpy skin disease virus (LSDV) is a DNA virus from the family *Poxviridae* that causes characteristic skin nodules in bovine species, such as cattle and buffalo. Because it affects the hide and can cause pyrexia, emaciation, abortions, and/or reduced milk yield in lactating cattle, it is a disease of agricultural and economic importance, and can devastate the livelihoods of farmers (1). The virus is primarily transmitted mechanically by biting arthropods, such as mosquitos, flies, and ticks, and its ability to survive in skin nodules for over a month facilitates rapid spread throughout a herd (2).

Historically, lumpy skin disease has been endemic throughout the majority of Africa. In late 2020 and early 2021, several epidemics in Asia were observed, involving China, Myanmar, and Vietnam (3). LSDV was first detected in domestic cattle in Cambodia in June of 2021. By September 2021, it had spread to all provinces throughout Cambodia and infected over 73,000 cattle and killed ~1,000 calves (personal communication GDAHP Sept 2021).

LSDV can also infect endangered and threatened wild bovids, such as banteng (*Bos javanicus*) and gaur (*Bos gaurus*). In neighboring Thailand, LSDV was detected in three gaurs in Kui Buri National Park in June 2021 and two bantengs in Huai Kha Khaeng Wildlife Sanctuary in August 2021 (4).

Globally, gaur have been listed by the IUCN as vulnerable since 1986 and banteng listed as endangered since 1996 (5, 6). There are estimated to be < 1,500 banteng in Cambodia and gaur populations are too small, fragmented, and infrequently observed to estimate. The majority of these species live in isolated protected areas in the Eastern and Northern Plains of the country.

Eradication and control of LSDV in domestic livestock relies on early detection, widespread vaccination, movement restriction, and removal of infected animals. Widespread vaccination with a homologous vaccine is paramount to a LSDV control program and no country has been able to eradicate LSDV without vaccination (3).

This case of lumpy skin disease in a free-roaming wild banteng in Cambodia was detected as part of WildHealthNet, an initiative in Cambodia, Laos, and Vietnam to develop national wildlife health surveillance networks to detect, investigate, and respond to high-consequence pathogens at the wildlife-livestock-human interface (7). The rapid spread of LSDV in domestic cattle in Cambodia coupled with the susceptible populations of banteng and gaur, facilitated the need for One Health collaboration and an integrated approach to manage and control this important disease at the wildlife-livestock interface. Through the network created under WildHealthNet, government and conservation stakeholders in the region were called to action to monitor banteng and gaur populations for clinical signs of LSDV, inform targeted vaccination strategies in livestock designed to prevent transmission of LSDV to vulnerable species of wildlife, and contribute to LSDV control in domestic cattle.

## Case description

**Figure 1:**
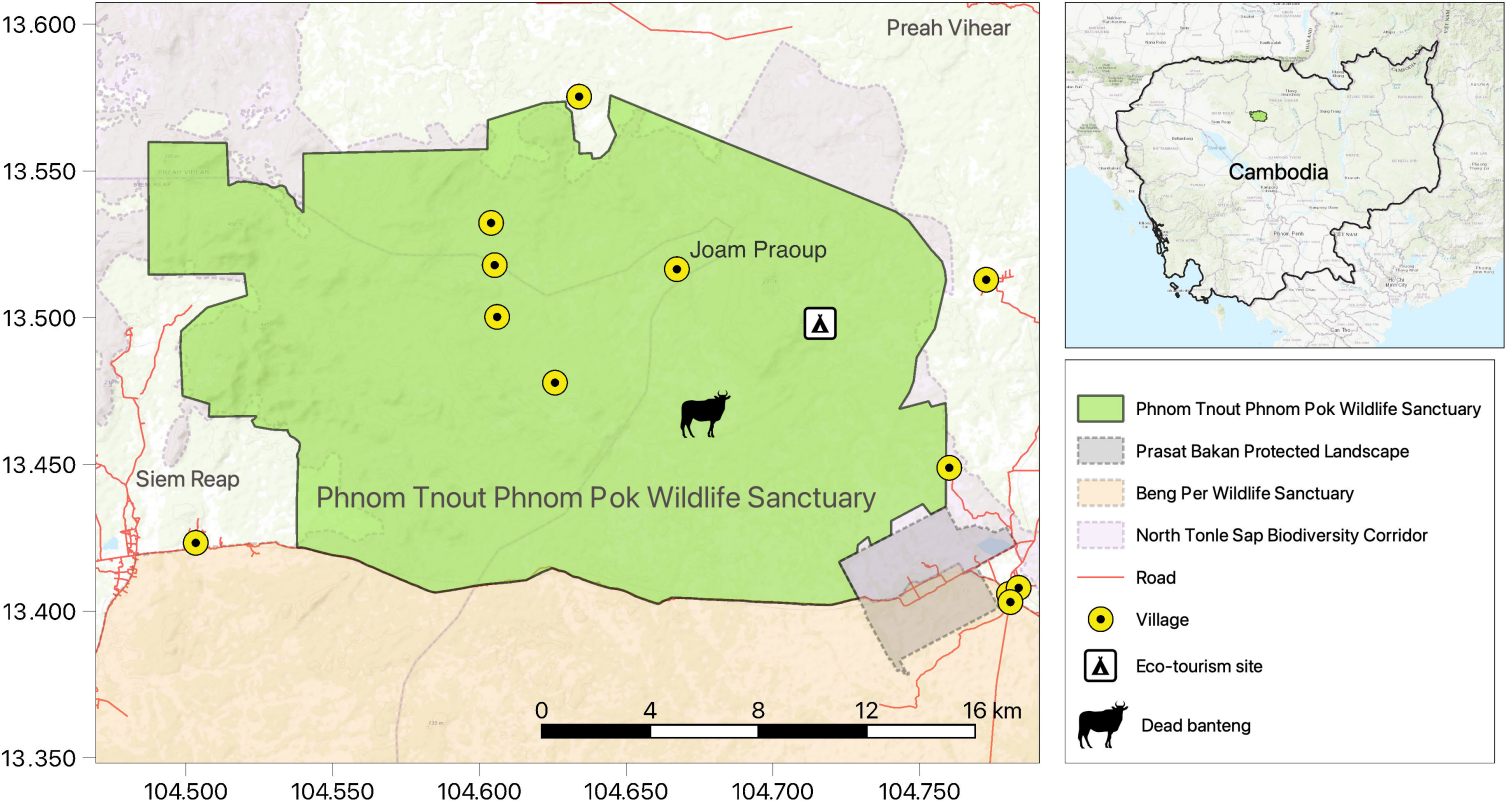
Map of Phnom Tnout Phnom Pok wildlife sanctuary with banteng case and village locations.

Phnom Tnout Phnom Pok wildlife sanctuary in Preah Vihear province is a 42,000 hectare dry dipterocarp forest that is home to a variety of endangered wildlife species including banteng, Sunda pangolins, elongated tortoises, and several primates, such as the Indochinese silvered langur (8). There are five villages within the wildlife sanctuary and several in the surrounding area (Figure 1). Protected areas in Cambodia are not fenced and domestic cattle are allowed to roam freely within them. It is estimated that 800–1,000 domestic cattle graze within the wildlife sanctuary and ~300 share the same area as banteng (personal communication Our Future Organization April 2023). Joam Praoup, a village within the wildlife sanctuary located 5.6 km from where the banteng was found, experienced a lumpy skin disease outbreak in domestic cattle as early as June 2021.

**Figure 2:**
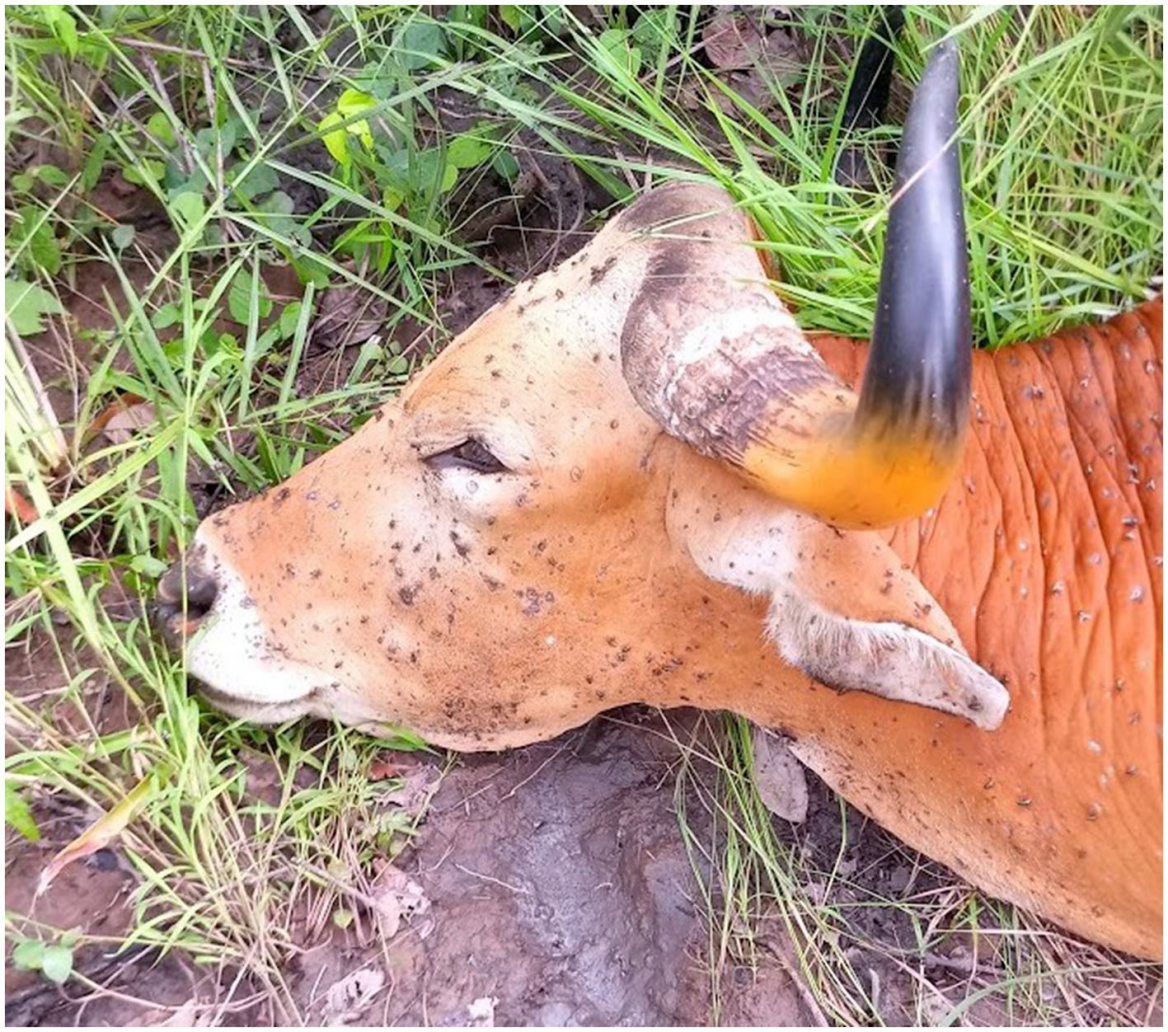
LSDV lesions on head and neck.

**Figure 3:**
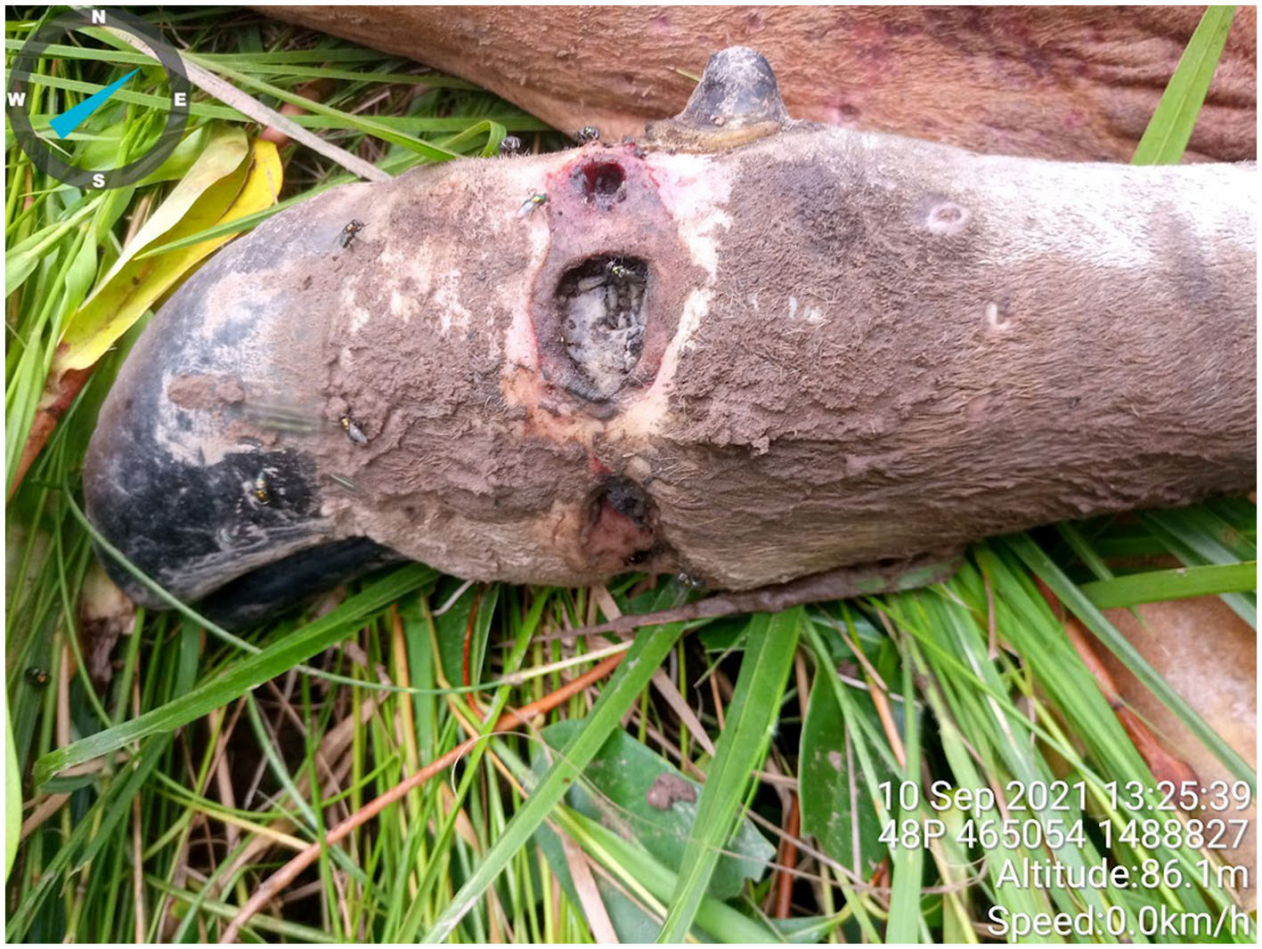
Necrotic snare wound.

On September 8, 2021, an adult male banteng was seen by community rangers in Phnom Tnout Phnom Pok wildlife sanctuary (Decimal degrees: *13.4665, 104.6765*). The banteng was thin, lethargic, and had multiple skin nodules on its head and flank (Figure 2). It also had a snare wound on its right front leg that appeared to be swollen and infected, causing lameness (Figure 3). Community rangers stayed with the animal to monitor its health and protect it from poachers. The banteng became progressively weaker and immobile, and eventually died on September 10 (Decimal degrees: *13.4672, 104.6771*).

Response actions were coordinated through the wildlife health surveillance network and government officials arranged for a Preah Vihear district veterinarian to travel to the site to collect samples. Whole blood and scabs from skin lesions on the head were collected. Following sample collection, the carcass was incinerated at the site.

The samples were sent to the Cambodian National Animal Health and Production Research Institute on September 13, 2021 and the scab samples were tested for LSDV via real-time-PCR following DNA extraction (Qiagen QIAamp^®^ DNA Extraction protocol) (9, 10). Results confirmed that the banteng was positive for LSDV.

## Response

Following the detection of LSDV in the banteng, conservation organizations, including Wildlife Conservation Society (WCS) and World Wide Fund for Nature (WWF), met with the Cambodian General Directorate of Animal Health and Production (GDAHP) to discuss a plan to prevent LSDV transmission to banteng and gaur. In order to protect these species from LSDV, the disease must be controlled in domestic cattle. Widespread vaccination, movement restrictions, and biosecurity in domestic cattle are essential for a LSDV control program. Unfortunately, resources and capacity for disease control in Cambodia are limited. At the time of the outbreak, GDAHP received 20,000 lumpy skin disease vaccines, however there are over 1.3 million susceptible cattle in Cambodia (personal communication GDAHP Sept 2021).

**Figure 4:**
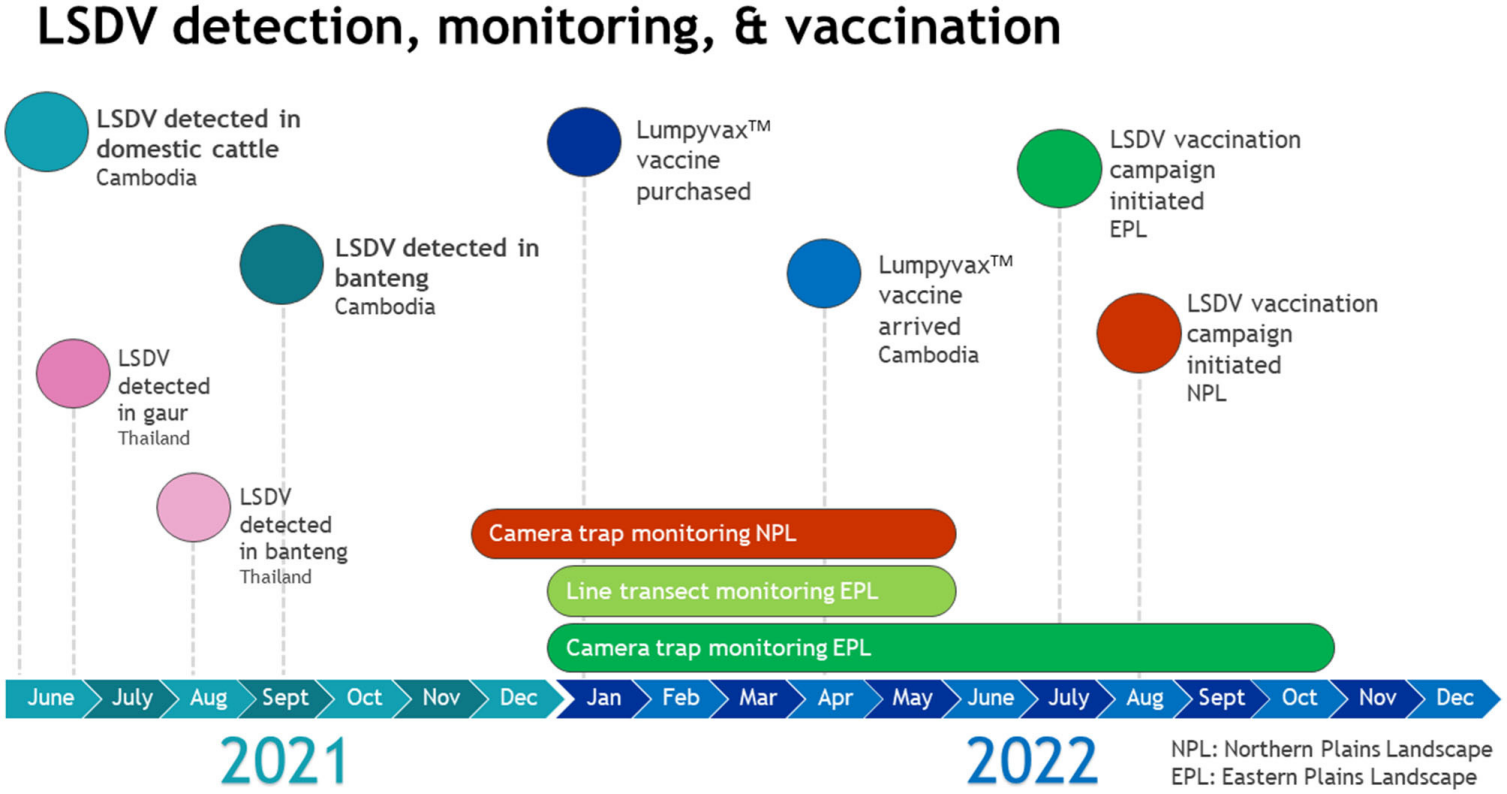
Timeline of LSDV detection, monitoring of wild ruminant populations, and vaccination campaign implementation.

A vaccination campaign was organized in coordination with GDAHP, provincial animal health departments, WCS, and WWF to augment the national LSDV vaccination effort. All parties agreed to conduct a ring vaccination of domestic cattle in and around the Eastern and Northern Plains Landscapes. 27,000 Lumpyvax^TM^ vaccine doses (100 doses/vial), needles and syringes were purchased in January 2022 by WCS Health program and Keo Seima REDD+ and WWF (Figure 4).

Lumpyvax^TM^ is a homologous live attenuated Neethling strain vaccine produced by Intervet in South Africa. It was approved for use in Cambodia by GDAHP based on preliminary efficacy studies completed internally in Cambodia at the start of the outbreak in domestic cattle (personal communication GDAHP April 2023).

The Eastern Plains Landscape (Srepok, Phnom Prich, and Keo Seima wildlife sanctuaries) in Mondulkiri province were chosen as priority protected areas to implement the vaccination campaign due to their importance for banteng conservation. It was agreed that all domestic cattle in and within a 20 km radius of the protected areas would be vaccinated for LSDV. A distance of 20 km surrounding each protected area was chosen as the radius for the ring vaccination zone according to known vector range and historical vaccination recommendations (11). All remaining vaccines would be designated for distribution to the Northern Plains Landscape (Kulen Promtep, Chhaeb, Preah Roka, and Phnom Tnout Phnom Pok wildlife sanctuaries) in Preah Vihear province to vaccinate domestic cattle in select villages in close proximity to known banteng populations.

The vaccination campaign was implemented in the Eastern Plains Landscape from July 21-December 28, 2022 and in the Northern Plains Landscape from August 20-October 12, 2022. Community awareness and vaccination campaign launch events were organized by GDAHP, in coordination with WWF and WCS, in Srepok wildlife sanctuary in the Eastern Plains, and the Preah Vihear Provincial Animal Health and Production Office in Chhaeb district in the Northern Plains, at the start of each vaccination campaign, respectively. These events aimed to raise awareness among local communities about the LSDV vaccination campaign and educate village animal health workers on vaccination procedures. Twenty thousand vaccine doses were distributed to animal health authorities in the Eastern Plains Landscape and 5,000 were distributed to authorities in the Northern Plains Landscape.

Post vaccination campaign reports confirmed a total of 14,226 domestic cattle and 2,646 water buffalo were vaccinated for LSDV in the Eastern Plains and 2,676 domestic cattle and 541 water buffalo were vaccinated in the Northern Plains. The remaining 2,000 vaccine doses will be distributed to both landscapes in May 2023 to complete a second phase of vaccination. GDAHP has committed to continue vaccination for LSDV for an additional 3 years (personal communication GDAHP April 2023).

Monitoring for clinical signs of LSDV in wildlife was conducted by forest ranger patrol teams and conservation research teams in Srepok, Phnom Prich, and Keo Seima wildlife sanctuaries. A distance-sampling based line transect survey was conducted in all three Eastern Plains protected areas over the dry season (Jan-May) of 2022. During this survey, research teams directly observed banteng on seven occasions (group size ranged from 1 to 5 individuals) in Srepok. In Phnom Prich one gaur was observed and banteng were observed on seven occasions (group size ranged from 1 to 6 individuals). No signs of LSDV were seen on any of the observed wild bovids. In Keo Seima, neither banteng nor gaur were directly observed.

Camera trapping was conducted in the core zone of Srepok January-May and July-October 2022, in the core and conservation zone of Phnom Prich February-May 2022, in Keo Seima March-August 2022, and Kulen Promtep December 2021-May 2022. In Keo Seima, banteng were observed on camera trap images on two separate occasions in March (group of four) and May (group of six) 2022. No visible signs of LSDV were observed on the images. Gaur were not observed in Keo Seima. Both banteng and gaur were observed on camera trap images in Phnom Prich and Sre Pok, and banteng only were observed in Kulen Promtep, however no visible signs of LSDV were observed.

## Discussion

This is the first case of LSDV reported in a banteng in Cambodia, adding to the earlier regional cases reported in Thailand. With widespread transmission of LSDV and prevalence of free-roaming domestic cattle in and around protected areas, this case epitomizes the imminent and incessant health-related threats to conservation at the wildlife-livestock interface.

In rapidly developing biodiversity-rich regions, habitat destruction, land-use change, and human encroachment into wild areas create overlapping habitats and novel interfaces with more frequent interactions between wildlife and domestic animals and increased opportunities for disease transmission. Wildlife is often blamed for spillover of diseases to humans (i.e., SARS-CoV) and domestic animals (i.e., HPAI, Nipah virus), however recognition of the risks of disease transmission to vulnerable and endangered populations already ravaged by habitat loss and wildlife trafficking is often neglected. Spillover of domestic animal diseases to wildlife represents a serious threat to conservation (12). In Mongolia, nearly 80% of the critically endangered Mongolian saiga antelope population were lost due to *Peste des Petits Ruminants* (PPR) virus, spread from sheep and goats (13). In Southeast Asia, 11 endemic wild pig species, including the Sumatran bearded pig *(Sus barbatus)*, are threatened by the widespread and catastrophic African swine fever virus epidemic in domestic swine (14, 15). In addition to primary hosts, the loss of critical keystone species due to disease transmission could have amplifying long-term consequences on the ecosystem, such as causing diminished food sources for endangered predators.

This case also highlights another formidable threat to endangered wildlife, the snaring crisis. Although the banteng developed lumpy skin disease, its snaring injury likely contributed to its inability to fight off the infection and eventual death. Wild cattle populations were severely depleted due to decades of hunting pressure historically, and currently wild bovids are severely threatened by the snaring epidemic in Southeast Asia. It is estimated that there are over 12 million snares in the protected areas of Cambodia, Laos, and Vietnam (16). Large wild bovids, including banteng and gaur, are often targeted for snaring due to the increased demand for wildlife meat, driven by wealthier classes in urban areas (17). Snaring is an enticing option for rural villagers to support their livelihoods, as snares are made from inexpensive readily available materials, such as cord and wire, and numerous snares can be deployed in a single outing (18). Often snares are not checked by the hunter who set them, leaving whatever indiscriminate species to perish due to starvation or infection from the wound (16). Despite snaring being illegal in Cambodia under the 2002 Forestry Law and 2008 Protected Areas Law, over the last decade there has been a rapid intensification of poaching, with detection rates of lethal traps by rangers increasing over 100-fold (19). Limited governance, lack of resources for law enforcement, and corruption enable the snaring crisis to persist and threaten wildlife populations.

The survival of a species is also considered at risk when confined to small, fragmented, isolated populations. Only one decade ago, the Eastern Plains in Cambodia held the largest population of banteng globally, with an estimate population size of 4,600 per IUCN with total global population estimated to be 4,000–8,000 (5, 20). The banteng population in two key protected areas within the Eastern Plains Landscape, Srepok and Phnom Prich wildlife sanctuaries, declined by a dramatic 72% in the decade between 2010 and 2020 based on a robust long-term distance-sampling based monitoring (19). By 2022, the estimated banteng population had further dropped by 89% compared to the baseline year (Groenenberg et al. unpublished data). In the adjacent Keo Seima wildlife sanctuary, line transect failed to detect any banteng in recent years despite extensive survey effort (21–23). Banteng in Phnom Tnout Phnom Pok wildlife sanctuary are estimated to be 46–119 individuals based on camera trap data, which makes it a small but globally significant population as there are only 6–8 subpopulations with over 50 animals (excluding Phnom Tnout Phnom Pok wildlife sanctuary) (8). Gaur are globally vulnerable and estimated to be 15,000–35,000 individuals worldwide (6). Cambodia harbors one of the most significant populations in the world, however surveys are currently too infrequent to estimate the population size. Evidence from other systems suggest that this case of LSDV in a banteng in Cambodia represents an additional threat to the conservation of wild ruminants in this region. We have seen that outbreaks of infectious disease can cause significant direct mortality when a pathogen enters an immunologically naive population of wildlife (13) and the presence of a pathogen circulating in a fragmented population of an endangered species can further increase extinction risk (24).

Mitigation measures to reduce disease transmission at the wildlife-livestock interface typically include biosecurity and movement control, however, these measures have limited application in wildlife populations. Vaccination of livestock in critical ecosystems is a potential intervention to prevent spillover of pathogens to susceptible wildlife, however, assessments of these types of interventions in peer-reviewed literature are limited. Recognition of the occurrence and impact of PPR in wildlife species, such as the critically endangered Saiga antelope (*Saiga tatarica mongolica*) in Mongolia, has recently been integrated into the FAO/WOAH PPR Global Eradication Programme with targeted PPR vaccination of domestic sheep and goats highlighted as critical to reducing pathogen transmission across the entire host community, including wildlife populations (25, 26). In Africa, there are disease prevention guidelines recommending immunization of protected area authorities, tourists, and other humans working in close proximity of great apes for childhood vaccines, including measles and polio, to prevent transmission of human pathogens to endangered apes (27). Direct vaccination of critically endangered wildlife is generally a last resort option. However, a canine distemper virus (CDV) vaccine was administered to foxes in Santa Catalina Island in California to successfully reestablish their population following a CDV outbreak thought to be spread by domestic dogs (28) and has been identified as a means of mitigating CDV risk to tiger populations in Asia (29).

The LSDV vaccination campaign of domestic cattle in and around protected areas in Cambodia was ultimately a successful initiative. Although we cannot conclude that the lack of detection of LSDV in banteng and gaur following the vaccination campaign was due to our efforts, it exemplified fruitful cross-sector collaboration to envision, draft, and implement a real-time animal emergency response and action plan. In addition, it increased awareness about LSDV and other infectious disease threats at the wildlife-livestock-human interface during the COVID-19 pandemic, and the downstream effects human behavior has on vulnerable wildlife populations in critical ecosystems. Perhaps most importantly, local livestock farmers and villagers were appreciative of our efforts to help protect their animals from LSDV. This opportunity for community outreach and engagement encouraged improvement of domestic animal health and fostered a relationship for future disease recognition and reporting.

Despite the campaign's successes, there were several limitations. While no additional cases of LSDV were detected in wild ruminants, cases could have gone undetected by line transect, camera traps, or in areas where monitoring does not occur. Although ring vaccination was attempted, undoubtably some cattle within the protected areas were missed, leaving opportunity for vectors to spread the virus. Of the 25,000 LSDV vaccine doses distributed, doses were inevitably lost due to vaccination handling challenges and storage logistics. Resources were limited and there were not enough vaccines for comprehensive ring vaccination to be completely effective in preventing transmission. Vaccination of domestic cattle alone is not enough to protect endangered banteng and gaur from the risk of extinction, however it is an important component of a holistic approach to address wildlife health and conservation issues.

Pathogen transmission at the wildlife-livestock interface is a complex, intricate, and frequently evolving challenge requiring intervention to prevent transboundary animal diseases from expanding their geographic range and spilling over to additional susceptible species. Spillover of livestock diseases to naïve fragmented vulnerable wildlife can have devastating consequences for endangered wildlife populations, biodiversity, and cascading effects on the ecosystem. Protecting these species from extinction requires a One Health approach and innovative integrated solutions. Vaccination is only a fraction of the solution and will not be effective without long-term commitment to conservation from government, decreased demand for wildlife meat, habitat protection, stronger law enforcement, and recognition of the role of wildlife in socioecological systems and transboundary disease response plans. The success of this multidisciplinary cross-sectional approach to detect LSDV in a banteng, activate surveillance for LSDV in critical wildlife populations, and implement a LSDV vaccination campaign in domestic cattle epitomizes the possibilities for unified One Health collaboration to protect endangered species from disease and other anthropogenic health threats.

## Data availability statement

The raw data supporting the conclusions of this article will be made available by the authors, without undue reservation.

## Ethics statement

The animal study was reviewed and approved by Wildlife Conservation Society Institutional Animal Care and Use Committee (vertebrate and invertebrate research). Written informed consent was obtained from the participant/patient(s) for the publication of this case report.

## Author contributions

AP drafted manuscript. AP, MG, and CA organized vaccination campaign funding and agreements with their respective institutions. SC, SrS, BD, and SD facilitated case reporting. ST facilitated case diagnostics. VN facilitated vaccination campaign implementation. VC, CH, SaS, and SK facilitated network communication. MG, CA, BD, and SD facilitated monitoring in their respective protected areas. SO and AF reviewed and edited manuscript. All authors contributed to the article and approved the submitted version.
